# Robustness, Generalizability, and Heterogeneity of Dynamic Networks of Psychopathology

**DOI:** 10.1007/s10608-025-10708-9

**Published:** 2026-02-10

**Authors:** Alberto Jover Martínez, Lourens J. Waldorp, Lotte H. J. M. Lemmens, Eiko I. Fried, Anne Roefs

**Affiliations:** 1 Department of Clinical Psychological Science, Maastricht University, Maastricht, The Netherlands; 2 Department of Psychological Methods, University of Amsterdam, Amsterdam, The Netherlands; 3 Department of Clinical Psychology, Leiden University, Leiden, The Netherlands

**Keywords:** Network approach, Dynamic networks, Psychopathology, Time series, MlVAR

## Abstract

**Purpose:**

The network perspective of psychopathology proposes that mental disorders arise from dynamic interactions between psychopathology-related variables. This study explored the robustness, generalizability, and heterogeneity of dynamic networks of psychopathology using Ecological Momentary Assessment data of 176 university students with varying degrees of subclinical psychopathology (M = 21.9 years, SD = 2.8; 83.3% female).

**Methods:**

Robustness—i.e., how precisely model parameters are estimated—of nomothetic networks was assessed via case-dropping bootstrapping. Heterogeneity was analyzed using the Individual Network Invariance Test (INIT) per pair of individuals. Generalizability (i.e., how much group-derived estimates reflect individual processes) was evaluated by comparing freely estimated idiographic networks (i.e., graphicalVAR) to idiographic networks where significant effects from the nomothetic network (i.e., mlVAR) were constrained to be present. This was done with 3 different γ values (i.e., 0.5, 0.25, and 0).

**Results:**

Results suggest that robustness was acceptable overall. Using a γ = 0.5, the nomothetic network generalized well to the majority of individuals, but not to a substantial minority. Specifically, the group model generalized well to 127 participants (73%) participants, but not to 47 (27%). However, with lower γ parameters, the group model generalized to less participants—161 participants (7.47%) at γ = 0.25 and 174 participants (0%) at γ = 0. Finally, evidence for relevant levels of inter-individual heterogeneity was found. Concretely, 3793 out of 14.878 (24.49%) pairs of individuals displayed different network structures according to the INIT test.

**Conclusions:**

This heterogeneity is a partial explanation of why treatments may not work for everyone and the individual networks provide a possible point of entry to determine more personalised treatments based on homogeneous groups. Recommendations to find such groups combining data-driven and theory-driven approaches with a focus on single-case research are discussed.

## Introduction

The network perspective to psychopathology offers an alternative view to the medical model of mental disorders (Borsboom, 2017; Fried, 2022; Scheffer et al., 2024; Bringmann et al., 2022). Rather than attributing the symptoms of mental disorders to underlying biological processes, as proposed by the medical model (Bruce, 2009; Deacon, 2013; Huda, 2021), this approach suggests that a mental disorder arises from dynamic interactions between symptoms (Borsboom, 2017). Such dynamic interactions occur between symptoms within and across diagnostic categories (Cramer et al., 2010) rendering the network approach transdiagnostic in nature. Besides symptoms, other variables have been proposed to be included in psychopathology networks, such as context, behaviors, or social interactions (Jones et al., 2017; Roefs et al., 2022). In the last decade, the network approach has made substantial progress, with increasing emphasis on dynamic and idiographic research (Brignmann, 2021; Mansueto et al., 2023), which has led to a number of studies using it in personalized psychotherapy (e.g., Hofmann et al., 2024, Hall & Luxemburg., 2023; Rubel et al., 2018). A persistent challenge for idiographic approaches is the generalizability of findings from nomothetic to idiographic levels (Fisher et al., 2018; Hamaker, 2012), a challenge that is particularly pronounced in network research due to the heterogeneity of idiographic networks (Beck & Jackson, 2020; De Vos et al., 2017; Levinson et al., 2022; Piccirillo & Rodebaugh, 2022; Reeves & Fisher, 2020). Importantly, such generalizability cannot be meaningfully evaluated without robust dynamic nomothetic models. Yet, to our knowledge, no studies have examined the robustness of such models. The overall goal of this study is to investigate three challenges of dynamic and idiographic network research. First, the robustness of nomothetic dynamic networks was examined. Second, the generalizability from nomothetic to idiographic dynamic networks was investigated in two different ways. Firstly, the heterogeneity of idiographic dynamic networks was investigated. Finally, the generalizability from nomothetic to idiographic temporal networks was studied.

### Goal One: Robustness of Nomothetic Dynamic Networks

Robustness, is defined as “the stability of statistical inference under variations of the accepted distribution models” (Shevlyakov & Vilchevski, 2002, p.7). In other words, robustness reflects whether the model estimation remains the same when data are obtained from different distributions, usually reflected in small variations of the data (e.g., bootstrapped samples, case-dropping). The robustness of certain types of network models has not been investigated yet. Network model estimates are susceptible to low power and violations of normality, both of which are common in psychological research (Blanchard et al., 2022; Epskamp, Borsboom et al., 2018a). Therefore, studying the robustness of networks is crucial to ensure the reliability of results (Blanchard et al., 2022; Epskamp, Borsboom et al., 2018a).

The robustness of cross-sectional networks (i.e., undirected network models estimated on *between*-individuals data) has been addressed frequently using case-dropping bootstrapping methods (Blanchard et al., 2022; Epskamp, Borsboom et al., 2018a). Specifically, new datasets are created by re-sampling cases with replacement, and the same network model is fit on each dataset. The parameters obtained from the fitted networks form a sampling distribution, and a bootstrapped confidence interval can be estimated (Epskamp, Borsboom et al., 2018a). Cross-sectional networks can be estimated robustly, but only with enough power and bootstrap permutations (Blanchard et al., 2022; Epskamp, Borsboom et al., 2018a). However, cross-sectional networks only inform about interactions between variables at one time point at the between-subjects level (Epskamp, Borsboom et al., 2018a).

Dynamic networks (i.e., network models estimated on temporal data) inform about the temporal relations between variables, which is crucial given the dynamic nature of network theory of psychopathology (Borsboom, 2017) and most psychological variables (Blanchard et al., 2022; Bringmann et al., 2022). Dynamic networks can entail a temporal network—reflecting temporal relations between measurement points—and a contemporaneous network showing the relations between the nodes’ residuals after fitting the temporal network. These residuals are theorized to reflect relations within measurement points (Epskamp et al., 2018). Different ways of studying robustness of dynamic networks have been suggested, such as case-dropping bootstrapping methods, or dropping blocks of data within-individuals, instead of full individual’s data to account for temporal dependencies (Epskamp et al., 2018). A study that estimated dynamic idiographic networks, pooled the idiographic networks together, and used a case-dropping bootstrapping method to examine the robustness of the pooled parameters. It was found that the contemporaneous networks were robust but the temporal networks were not (Lazarus et al., 2021).

Otherwise, the robustness of dynamic networks has been neglected. This could be because researchers prefer to estimate nomothetic dynamic networks instead of pooling the idiographic networks of different participants unless the sample is homogeneous (De Vos et al., 2017). However, the robustness of multi-level dynamic networks has not been studied to our knowledge because bootstrapping methods for such models are very computationally demanding (Bringmann et al., 2013). In the present study, the robustness of multi-level Vector Autoregressive model (Epskamp et al., 2018) of transdiagnostic psychopathology was studied.

### Goal Two: Generalizability from Nomothetic to Idiographic Networks

The generalizability of nomothetic models to idiographic models—that is, whether group-derived estimates reflect idiographic processes (Fisher et al., 2018) —has been overlooked (Hamaker, 2012). Most research on the network approach is mostly carried out at the nomothetic level, ignoring if the studied processes apply to specific individuals and potentially compromising clinical utility (Molenaar, 2004; Molenaar & Campbell, 2009). This is especially problematic given that network research shows that idiographic networks are highly heterogeneous.

#### Goal 2a: Heterogeneity of Networks of Individuals

Investigating the heterogeneity of idiographic networks is crucial for understanding the generalizability from nomothetic to idiographic networks. The lack of generalizability of parameters derived from groups to individuals could be due to high heterogeneity. That is, parameters derived from a group consisting of very different individuals will not represent all individuals well. Moreover, understanding the degree of heterogeneity of networks is critical for the development of personalized interventions (Hoekstra et al., 2023).

Previous research suggests that idiographic networks are heterogeneous (Beck & Jackson, 2020; De Vos et al., 2017; Levinson et al., 2022; Piccirillo & Rodebaugh, 2022; Reeves & Fisher, 2020). However, this research relied on visual inspection of estimated networks to make inferences about the observed heterogeneity. Moreover, these studies attributed all heterogeneity to individual differences, ignoring sampling variability and limited power to properly assess inter-individual heterogeneity (Hoekstra et al., 2023). The present study investigates heterogeneity by examining differences in the network structure of each pair of individuals. To do so, we use a recently developed statistical test rather than relying on visual inspections.

#### Goal 2b: Generalizability from Nomothetic to Idiographic Networks

To investigate within-individual relationships among variables in a network, researchers typically estimate dynamic networks. However, researchers investigating within-individual relationships use methods that are not idiographic enough. For example, studies estimating one idiographic network per participant frequently pool such networks together. Pooled results do not represent the different individuals in the group sufficiently unless the group is homogeneous (i.e., the individuals are similar), but that is rarely the case (De Vos et al., 2017; Molenaar & Campbell, 2009). Another approach taking within-individual variation into account are multilevel models such as mlVAR models. However, multi-level models like mlVAR rely heavily on the between-subjects parameters (i.e., *shrinkage*; Epskamp et al., 2018). Thus, despite these attempts to approach the individual level, mlVAR relies heavily on group-based parameters.

Assuming that nomothetic results apply to all individuals poses a threat to social and medical sciences as there is evidence showing that this is likely not the case (Fisher et al., 2018; Hamaker, 2012). Given the clinical utility of knowledge of idiographic processes, researchers should study the generalizability of nomothetic results to the idiographic level (Fisher et al., 2018). In the present study, a freely estimated idiographic network model (hereon termed unconstrained model) and an idiographic network model where the parameters that were significant in the nomothetic network model (hereon termed constrained model) were estimated for each individual. After that, the difference in the goodness of fit was estimated to examine which model has a better fit. If most individuals display a better fit in the unconstrained model, it suggests that the nomothetic network does not generalize optimally.

## Method

The data used in this present study were collected in a study pre-registered with AsPredicted (https://aspredicted.org/79X_XY5) under registration number 62825. The analyses code is available in https://osf.io/jgqmu/overview. Requests for access to the data should be directed to fpn-nsmd-data@maastrichtuniversity.nl. If researchers want the data to check the analyses included in this paper, this will usually be allowed. Other requests will be reviewed promptly on a case-by-case basis. The study received approval from the ethical review board of the Faculty of Psychology & Neuroscience of MaastrichtUniversity.

### Participants

A group of 322 participants started the study, 288 completed the baseline questionnaire, 262 began the EMA protocol, and 238 reached the final week of the protocol. Participants who completed at least 50% of the EMA-surveys (*n* = 192) were included in the analyses to increase the number of datapoints per participant with the objective of increasing the estimates’ reliability. For more information about dropout see Jover Martínez et al. (2025). Due to lack of variance in at least one variable of the model, 16 participants needed to be removed from the analyses. The average age of participants was 21.9 years (*SD* = 2.8), 83.3% were female (*n* = 140), and 21.6% (*n* = 37) reported having been diagnosed with a mental disorder at some point in their lives. At the beginning of the study, only three participants were undergoing treatment for a mental disorder. The average number of answered surveys was 162.92, average compliance was 75.12% (SD = 12.55%), and the maximum was 97.73%. Importantly, relevant demographic information was not collected in this study.

### Procedure

For a complete description of the procedure of the study (e.g., recruitment), see Jover Martínez et al. (2025). The study consisted of a baseline questionnaire including standardized measures of psychopathology, an EMA practice day, and an EMA study that lasted 28 days during which different types of surveys were triggered with different frequencies. Specifically, there was a morning survey, and an evening survey triggered once per day, a weekly survey triggered once per week, and a momentary survey triggered 8 times per day (one of them together with the morning survey). For the present study, only momentary surveys were used (i.e., surveys triggered 8 times per day). Therefore, participants could answer up to 224 data points. Participants were rewarded based on the number of surveys they answered. Moreover, different surveys were rewarded differently. For example, the first survey of the day, which included items about sleep, had a reward twice as high as a regular survey.

Surveys were triggered semi-randomly within time windows of 1 h, 37 min, and 30 s. Moreover, surveys were triggered following a normal distribution (i.e., the chances of a survey triggered in the middle of the time interval were maximized) and the start of the daily triggers was adapted to participants’ usual waking times - see Jover Martínez et al (2025). All momentary surveys contained the same items. However, depending on the answer to the baseline questionnaire, some items were not triggered. For example, “Did you smoke since the last beep?” was only triggered if the participant indicated to be a smoker in the baseline questionnaire. Similarly, some items were contingent on answers to previous questions. For example, “What do you crave?” was only triggered when answering positively to the item “At this moment, I experience cravings”. All items were scored on Likert scales ranging from 1 to 7. The first moment (i.e. morning and first momentary surveys) expired after 45 min, and the others after 20 min.

Push notifications were sent when a survey was triggered, and 12 min before the expiration of the survey. An extra push notification for the first survey was sent 30 min before expiration. Every approximately 7 days, participants received emails updating them on their potential rewards if they continued to maintain their current response rate. In cases of non-compliance, participants were contacted to determine the reason, and if issues were identified, efforts were made to find suitable solutions.

### Measurements

For an overview of all the items included in the EMA protocol see Jover Martínez et al. (2024a). These items were selected based on focus groups with clinical psychologists and research experts on different mental health problems to ensure that they reflected a broad spectrum of psychopathology and were clinically relevant Jover Martínez et al. (2024a). In the current study, only a selection of items from the momentary surveys were used to estimate the following variables: positive affect, negative affect, somatic negative affect, self-esteem, enjoyment of activities, enjoyment of social activities, sense of control, concentration, worry, and impulsivity. Some of these variables were averages of a few items (e.g., positive affect, or negative affect). These variables were used as nodes in all the networks presented in this paper. For the specific variable configuration see Jover Martínez et al. (2025).

## Analysis

All analyses were performed using R 4.2.2 (R Core Team, 2024) and consisted of Vector Autoregressive (VAR) models. VAR models are linear time series models in which each variable at a previous time point is regressed on itself and all other variables in the model at a later time point (Brandt & Williams, 2007). For all models in the present study, lag-1 models—i.e., model estimating effects between *t − 1* and *t* were used -. VAR models for *n* = 1 (i.e., idiographic models) were estimated using the package *graphicalVAR* (Epskamp et al., 2018). This model provides two networks, a *temporal* network showing the temporal effects between variables, and a *contemporaneous* network showing the relations between the nodes’ residuals after fitting the temporal network. Such residuals are theorized to include relations that occur at a faster time scale than the relations captured in the temporal network (Epskamp et al., 2018a, b) Moreover, mlVAR models for *n* > 1 (i.e., nomothetic models) were estimated using the *mlVAR* package to account for the nestedness of the data. Sequential univariate multilevel estimation with orthogonal estimation of the random effects was used in mlVAR because it is recommended for networks with more than five nodes (Epskamp et al., 2018).

### Goal One: Robustness of Dynamic Networks

The robustness of temporal and contemporaneous networks estimated with mlVAR was studied. To study robustness of mlVAR, a non-parametric bootstrapping method was used where 75% of participants were sampled with replacement in 100 permutations. In each iteration, an mlVAR model was fitted. Usually, 500 permutations lead to accurate inference in bootstrapped analyses (Davison & Hinkley, 1997). However, any cutoff in the number of permutations is arbitrary (Haslbeck et al., 2025), and 500 permutations would be too demanding for the estimation of mlVAR models (Bringmann et al., 2013).

After the bootstrapping routine was completed, a sampling distribution for every relationship between the network’s nodes was created. This sampling distribution was used to estimate bootstrapped means, and bootstrapped Confidence Intervals (CI) based on the 0.025 percentile and 0.975 percentile. Moreover, the number of iterations that each effect in the networks was significant was estimated. In the temporal network, the relations are directed and include effects of each node with themselves. Therefore, *n*^2^ relations are estimated. In this study there were 10 nodes, which results in 100 relations. In the contemporaneous network the relations are undirected and do not include effects of the nodes with themselves. Therefore, only (*n* x (*n* − 1))/2 relations are estimated. In this study there were 10 nodes, which results in 45 relations for the contemporaneous network.

#### Goal 2a: Heterogeneity of Networks of Individuals

The heterogeneity of idiographic networks was studied by means of the recently developed Individual Network Invariance Test (INIT; Hoekstra et al., 2024). The INIT assesses the similarity of two graphicalVAR (Epskamp et al., 2018a, b) network structures. GraphicalVAR estimates a temporal and a contemporaneous network. INIT compares a heterogeneous model and a homogeneous model. The heterogeneous model allowed the network relations to vary between networks, and the homogeneous model kept the network relations constant across networks. Afterwards, the homogeneous model’s Akaike Information Criteria (AIC) was subtracted from the heterogeneous model’s AIC (AIC_heterogeneous_ − AIC_homogeneous_). Lower AIC values indicate better fit, which means that, after the subtraction, negative values indicated a better fit for the heterogeneous model, indicating that individuals were different, and positive values indicated a better fit for the homogeneous model, indicating that individuals were not different. In the present paper, all relations in the models were estimated (i.e., the models were saturated), and a comparison for every pair of individuals was performed. Given that estimation and convergence problems required the removal of 3 participants from the heterogeneity analyses, the final sample consisted of 173 individuals. A total of (*n* × (*n* − 1)/2 comparisons were performed, leading to 14.878 comparisons. Both the temporal and the contemporaneous networks were included in these comparisons.

#### Goal 2b: Generalizability from Nomothetic to Idiographic Networks

First, an mlVAR model was estimated for the whole sample to estimate the nomothetic model. Second, a graphicalVAR model was estimated per individual (i.e., an idiographic model) without any constraints (i.e., unconstrained model). Third, a constrained graphicalVAR model was estimated per individual where only the effects that were significant in the nomothetic model were estimated (i.e., constrained model). Specifically, the effects of the temporal and contemporaneous networks of the nomothetic model were constrained in the temporal and contemporaneous structures of the graphicalVAR model. Finally, the Estimated Bayesian Information Criterion (EBIC) of the constrained model was subtracted from the unconstrained model to determine which model had better fit (EBIC_unconstrained_ − EBIC_constrained_). Lower EBIC indicates a better fit, which means that, after the subtraction, negative values indicated a better fit for the unconstrained model, and positive values indicated a better fit for the constrained model. EBIC uses a hyperparameter γ, which determines how strongly the EBIC favors simpler models with fewer edges (Foygel & Drton, 2010). The choice of this hyperparameter is somewhat arbitrary and depends on the value placed on caution—i.e., stricter models with fewer false positives—versus discovery—i.e., richer models with more detected edges (Dziak et al., 2012). Therefore, three different γ values were used in the analyses: 0.5, 0.25, and 0.

In the constrained model, the effects from nomothetic network were always included in each idiographic model, to test how well the nomothetic network fits each individual’s data. This means that effects were included that might not have been present if the model had been estimated without constrains. In other words, false positives might have been forced. Therefore, the percentage of effects close to 0 was estimated for the constrained model as an indicator of potential false positives. Specifically, the percentage of effects between − 0.1 and 0.1, and between − 0.05 and 0.05 were estimated. That percentage was also estimated in the unconstrained model to examine how different the percentage between models was.

## Results

### Goal One: Robustness of Dynamic Networks

**Fig. 1:**
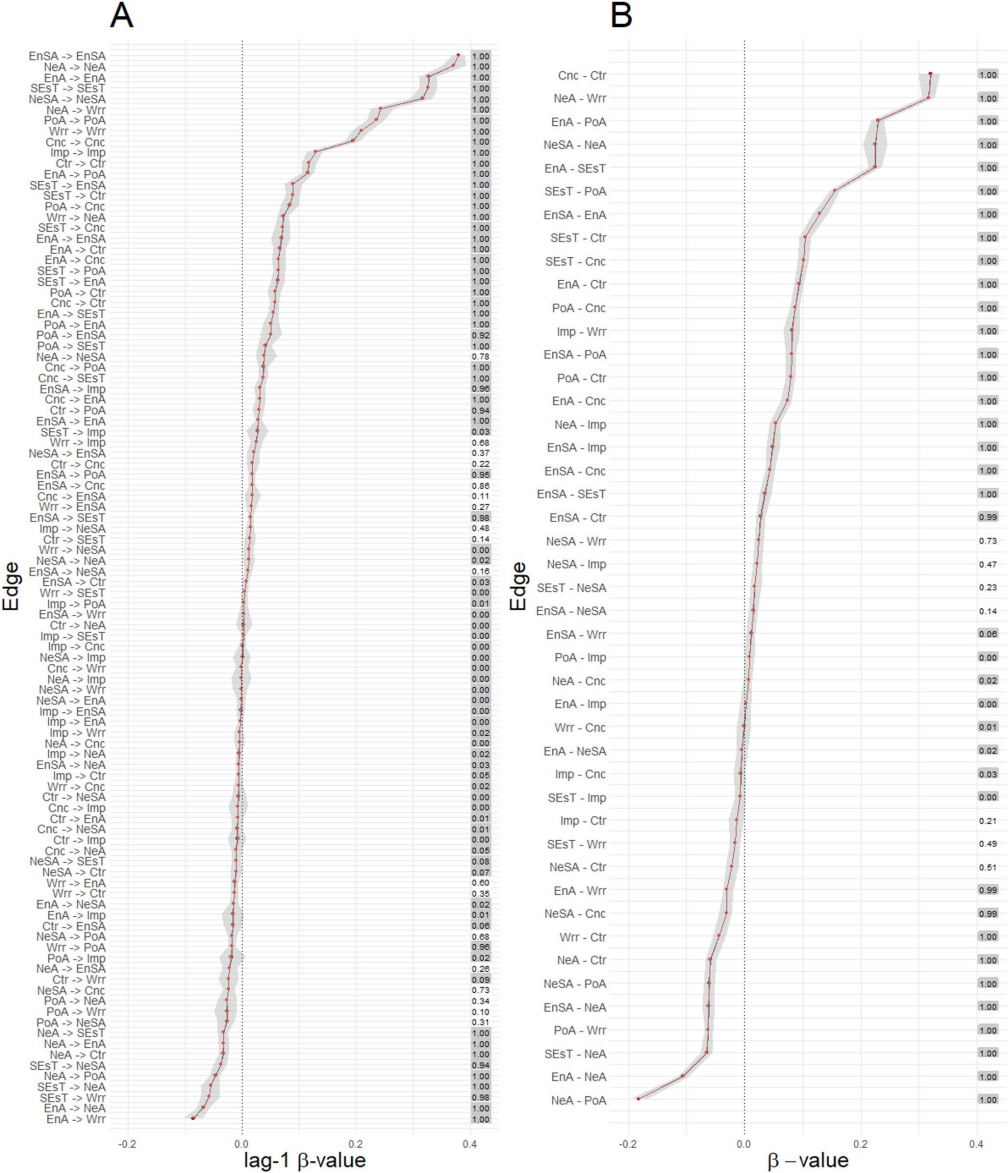
Visualization of robustness analyses. *Note*: **A** Robustness results for temporal networks, **B** Robustness results for contemporaneous networks. Red dots represent the values for the group model means, and black dots for the bootstrapped means. The gray shadows represent the CI, and the numbers in boxes the proportion of iterations each effect was significant. Grey boxes represent effects that were significant in 90% of iterations or more (i.e., consistently observed), or 10% of iterations or less (i.e., consistently not observed), whereas white boxes represent effects that were significant in between 11% and 89% of iterations. Ctr = Sense of control, Cnc = Concentration, Wrr = Worry, Imp = Impulsivity, PoA = Positive Affect, NeA = Negative Affect, NeSA = Negative somatic affect, SEsT = Self-esteem, EnA = Enjoyment of Activities, EnSA = Enjoyment of social activities

Figure 1 provides a visual representation of the robustness results, which overall suggest that the mlVAR estimates were robust. The group means and bootstrapped means were very similar for all networks, meaning that estimates were robust to sampling variation. The confidence intervals were narrow for the temporal and contemporaneous networks (panels A and B of Fig. 1 respectively). Regarding the significance of the effects, 17 relations (17%) for the temporal network, and 7 effects for the contemporaneous network (15.6%) were not significant consistently (i.e., such effects were significant only between 10% and 90% of the iterations). Evidence for 24 effects in the temporal network (24%), and 6 in the contemporaneous network (13.3%) was not consistently absent (i.e., the confidence intervals contained 0).

#### Goal 2a: Heterogeneity of Networks of Individuals

**Fig. 2:**
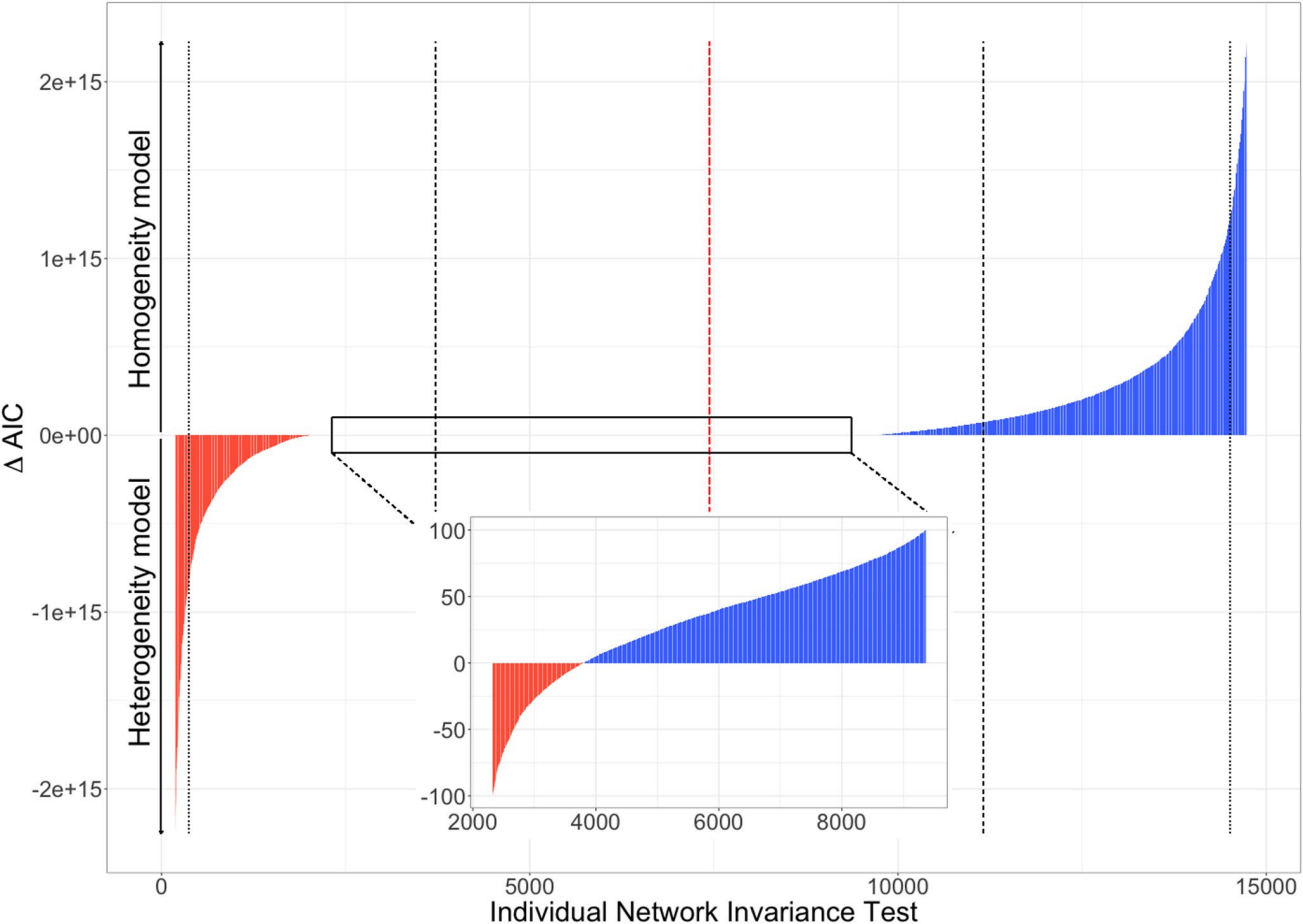
Distribution of AIC differences. *Note*: AIC = Akaike Information Criterion. ΔAIC = AIC_heterogeneous_ − AIC_homogeneous_. A positive ΔAIC value means that the homogeneous model has a better fit. A negative ΔAIC value means that the heterogeneous model has a better fit. The red dashed line represents the median, the black dashed lines the 0.25 and 0.75 quantiles, and the dotted dashed line the quantiles 0.025 and 0.975. The zoomed-in plot represents the ΔAIC scores between − 100 and 100. Each column represents a comparison between networks of two individuals

Figure 2 represents the difference in AIC for each comparison between pairs of individuals. Overall, the figure shows that the homogeneity model (constraining network structures of two individuals to the same structure) had a better fit in most comparisons than the heterogeneity model (allowing network structures of two individuals to be different). Specifically, in 11,085 (74.51%) of the model comparisons, the homogeneous model had a better fit, and in 3,793 (24.49%) the heterogeneous model had a better fit. 330 comparisons (2.21%) had a standard deviation above 1 or below-1 and were removed from the plot to improve legibility.

#### Goal 2b: Generalizability from Nomothetic to Idiographic Networks

**Fig. 3:**
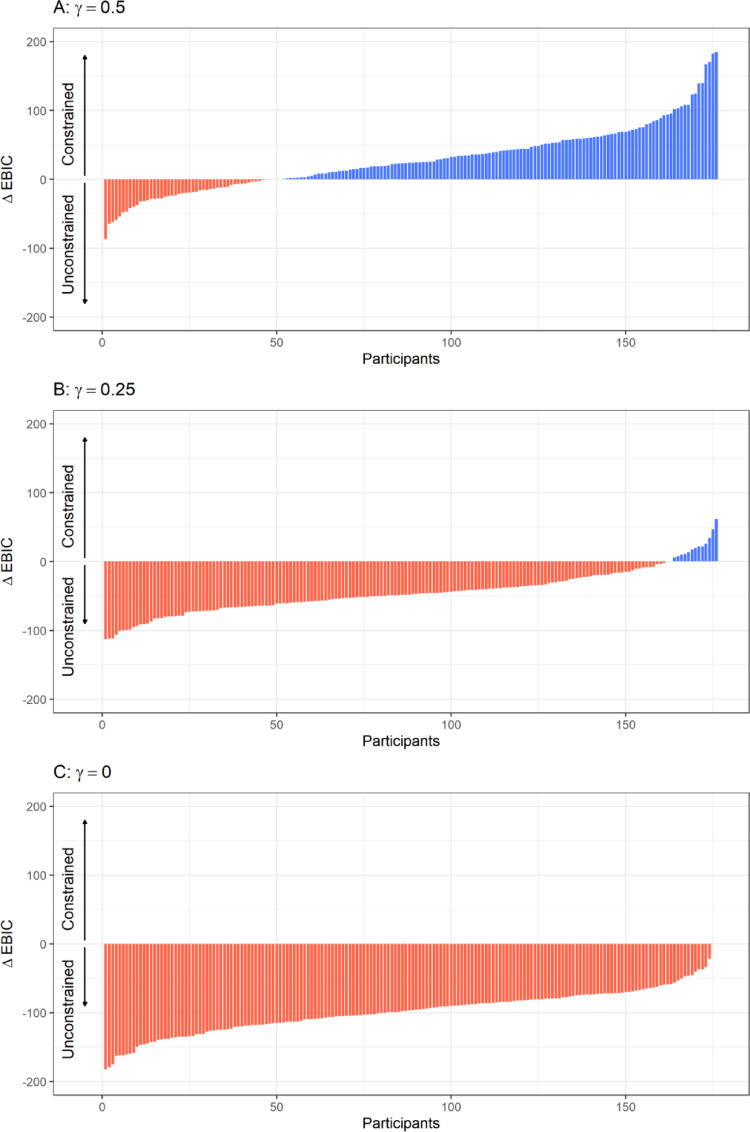
Distribution of EBIC differences. *Note*: EBIC = Extended Bayesian Information Criterion. ΔEBIC = *EBIC*_*unconstrained*_ − *EBIC*_*constrained*_. Red bars indicate a better fit for the unconstrained model and blue bars indicate a better fit for the constrained model. Each column represents a participant

Estimation and convergence problems for 2 participants required their removal from the generalizability analyses, leaving a final sample of 174 individuals. Panels A, B, and C of Fig. 3 show the difference between the constrained and unconstrained models on the EBIC when γ = 0.5, 0.25, and 0 were used, respectively. When γ = 0.5, the constrained model had a better fit for 127 (72%) participants, and the unconstrained model had a better fit for 47 (27%) participants. Together, this suggests that the nomothetic model generalized well to most idiographic models in this condition. However, 51.56% of the constrained model’s parameters were between − 0.1 and 0.1, whereas only 35.17% of the unconstrained model’s parameters were within that range. Moreover, 28.63% of the constrained model’s parameters were within − 0.05 and 0.05, but only 14.17% of the unconstrained model’s parameters were within that range. This indicates that due to the forcing of parameters in the constrained models, some of the parameters might have been false positives.

Moreover, reducing γ led to markedly different results. When γ = 0.25, the number of participants showing a better fit for the constrained model dropped to 13 (7.47%), and reached 0 (0%) when γ = 0. Conversely, the number of participants showing a better fit for the unconstrained model increased to 161 (92.53%) at γ = 0.25 and 174 (100%) at γ = 0.

## Discussion

The nomothetic dynamic networks of the participants remaining after excluding participants with lower response rates demonstrated robustness. Regarding the generalizability results, the constrained model fitted well in most individuals (73%) when γ = 0.5, but it did not when γ = 0.25 (7.47%) or γ = 0 (0%). Moreover, the number of participants for whom the constrained model fitted well with γ = 0.5 might in reality be smaller becausefalse positives might have spuriously improved the goodness of fit of the constrained model. Finally, the heterogeneity analyses showed that estimated networks differed across a number of individuals, which indicates the presence of heterogeneity.

### Robustness of Dynamic Networks

The presented bootstrapping analyses showed that, with the exception of a few edges, the presented mlVAR’s temporal and contemporaneous networks were robust. It cannot be concluded that mlVAR models are robust in general because the robustness of mlVAR models depends on multiple things. For example, the true network, specific processing or analytical decisions, the number of between- and within-individual data points, or the number of nodes included in the model (Mansueto et al., 2023). Therefore, the presented results only apply for models estimated on a similar number of nodes, datapoints and participants. Researchers interested in using mlVAR models are encouraged to perform the presented analyses as exploring the robustness of effects is vital given how frequent underpowered studies and violations of the normality assumption are (Blanchard et al., 2022).

Moreover, researchers are encouraged to explore robustness in different ways. In the present study, robustness was studied by introducing variability at the between-individual level (i.e., in each iteration a proportion of individuals were removed). However, future research could remove chunks of data within-individuals, or a combination of chunks of data within-individual and whole individuals (Epskamp, 2020).

### Generalizability from Nomothetic to Idiographic Networks and Heterogeneity of Individual Networks

The generalizability analyses were markedly different depending on the γ parameter. The constrained model fit well for most individuals only when γ = 0.5. This good fit might be due to a similar data-generating mechanism across individuals (i.e., individuals share the same processes). Moreover, the results suggest that mlVAR models effectively capture and summarize these mechanisms. However, follow-up analyses suggests that the apparent goodness of fit for the constrained model might be spurious. In the constrained model, the effects of the nomothetic model were forced to assess their fit with each individual’s data, which may have resulted in false positives. This is suggested by the higher proportion of very small but significant effects (i.e., between − 0.05 and 0.05) in the constrained model. These effects likely would not have been significant without being forced. Including these effects was necessary to evaluate the generalizability of the nomothetic model to idiographic models, which might have artificially improved the constrained model’s fit, leading to an unfair comparison.

However, when γ = 0.25 and γ = 0, the constrained model did not fit well. This sensitivity is conceptually expected. Stronger regularization (higher γ) retains only the most robust edges—relations that tend to generalize across individuals—whereas weaker regularization (lower γ) allows weaker and more person-specific edges to enter the model. As a result, a higher γ emphasizes shared processes, while a lower γ increases apparent heterogeneity. Therefore, while the nomothetic model appears generalizable to most individuals, this is only the case at γ = 0.5. With lower γ values, the opposite holds—i.e., the nomothetic model does not generalize well.

Idiographic VAR models require sufficient time points to reliably estimate temporal and contemporaneous edges, especially when networks contain many nodes. Recent work (Zhang et al., 2025; Mansueto et al., 2023) has shown that models with 8–10 nodes may need considerably more than 200 observations to avoid overfitting and low power. Our idiographic models should therefore be interpreted cautiously. Because Goals 2a and 2b focused on relative differences between constrained and unconstrained networks—rather than precise estimation of individual edges—we emphasize that the results reflect patterns of model fit rather than exact edge values. However, as Zhang et al. (2025) note, predictive accuracy can still be limited when the number of datapoints is modest. Therefore, conclusions regarding heterogeneity (Goal 2a) and generalizability (Goal 2b) should be understood in light of this methodological constraint.

The results of the heterogeneity analyses are in line with the generalizability analyses. While the majority of comparisons favoured the homogeneity model, a number of individuals displayed different network structures. This suggests inter-individual heterogeneity due to different data-generating mechanisms (i.e., individuals display different processes). Together with the generalizability results this suggests that while the nomothetic model was generalizable to most individuals at γ = 0.5, those individuals who were not well represented by this model may have differeddue to inter- individual heterogeneity. Notably, work on minimally sufficient sets of symptoms suggests that heterogeneity may partly reflect redundant pathways rather than truly distinct processes (Fisher, 2025). Future work could integrate this perspective into network modeling by identifying minimally sufficient sets of edges as a function of a clinical benchmark. This approach may reduce apparent heterogeneity and highlight core relations driving individual differences.

A relevant avenue for future research is establishing a fairer comparison between the unconstrained and the constrained model. For example, this could be to take out the smallest effects in the constrained model until both the unconstrained and the constrained model have the same number of significant effects. However, it could be argued that with this approach the constrained model does not represent the true constrained model anymore. Alternatively, simulation studies could replicate the presented generalizability analyses with simulated data of individuals coming from the same model (homogeneous individuals) and from different models (heterogeneous individuals). Such a study would show what is the expected EBIC difference or what percentage of individuals display a better fit for each model when the sample is homogeneous and when the sample is heterogeneous. Based on these results it will be easier to draw conclusions if these analyses are applied to real data.

The limited generalizability of the nomothetic model together with the heterogeneity results show the urgency of finding criteria to define homogeneous groups. Such homogeneous groups are needed because nomothetic findings from heterogeneous groups might not be applicable to the individuals composing such group. Only when nomothetic research is carried out using homogeneous groups the findings are applicable to all individuals in that group (Molenaar & Campbell, 2009). Usually, diagnostic criteria are used to create homogeneous groups based on a diagnostic label, such as Major Depressive Disorder (MDD). However, it has been shown that there is large inter-individual heterogeneity is symptom profiles among people sharing the same diagnosis (e.g., MDD; Fried et al., 2020; Fried & Nesse, 2015). More specifically, the unique MDD profiles based on symptom combinations were studied in 3703 depressed outpatients. The most frequent profile was shared by only 2% of individuals, and 14% of individuals had unique profiles not shared by anybody else. Finally, 86.2% of profiles were shared by 5 individuals or fewer. These findings align well with the results of the current study, similarly pointing to large inter-individual heterogeneity, even when the people in the group share the same diagnosis. However, studying the homogeneity of individuals displaying sets of symptoms that are minimally sufficient to meet a clinical benchmark (Fisher, 2025) might help find more homogeneous groups.

Identifying more homogeneous groups may eventually improve the effectiveness of psychological treatments. Currently, the success of psychological treatments is modest overall (Holmes et al., 2018; Reynolds et al., 2012), and people who recover relapse frequently (Clark, 2018; Layard & Clark, 2015; Roefs et al., 2022). That might be because treatments are developed based on mechanistic research carried out at the nomothetic level. This is not optimal as nomothetic research is only generalizable to all the individuals in a sample under strict conditions that are rarely met, such as homogeneity of the sample (Molenaar & Campbell, 2009). Therefore, it is increasingly urgent to bring an idiographic focus to research, and psychological interventions. Methods such as network analysis and ecological momentary assessment (EMA) show promise in this regard, as evidenced by recent research highlighting their feasibility in clinical practice and utility for creating idiographic models tailored to individuals (Frumkin et al., 2021; Piccirillo & Rodebaugh, 2019). Moreover, finding homogeneous groups is vital for nomothetic research to be insightful about individuals (Molenaar & Campbell, 2009) and, consequently, finding more effective psychological treatments for individuals. Integrating findings from idiographic research with traditional nomothetic approaches can lead to a more comprehensive understanding of psychological processes and may help finding homogeneous groups. First, nomothetic bottom-up approaches, such as clustering methods that align with network analysis methods, may be able to identify more meaningful subgroups with similar network structures. Examples of such clustering methods are the subgrouped chain graphical VAR (scGVAR; Park et al., 2024) or the Latent Class Vector Autoregressive Models (Ernst & Haslbeck, 2025). However, the number of subgroups using scGVAR needs to be predefined. Second, idiographic top-down research could investigate possible indicators of homogeneous groups such as theory-driven mechanisms from a network perspective. If specific mechanisms are relevant for some individuals and not for others, such mechanisms may be a good criterion for identifying a homogeneous group. For example, individuals’ mood or anxiety problems may arise from different coping mechanisms (e.g., avoidance or substance abuse) that are motivated by different thoughts, and reinforced by different appraisals. Therefore, different coping mechanisms, appraisals of situations, or thoughts might be relevant criteria to identify homogeneous groups. Top-down approaches can be used with small samples to validate potential mechanisms that might be relevant indicators of homogeneous groups. There are a number of ways to validate such mechanisms from a network approach using idiographic research. For example, investigating whether the networks of people with similar coping mechanisms, thoughts and appraisals display the same structure. Analyses such as the INIT (Hoekstra et al., 2024), mlVAR group comparison (Haslbeck et al., 2025), or single case experimental designs (Vlaeyen et al., 2022) could assist with this purpose. Once some potential homogeneous groups have been identified, the data of a number of different homogeneous groups can be fed to scGVAR. Since the number of groups is known a priori, scGVAR can confirm whether a clustering solution with the defined number of groups has a good fit. Moreover, it can be confirmed whether the individuals that were thought to belong to the same group cluster together. It is important to note that both bottom-up and top-down approaches may benefit greatly from using lower-level networks (Jover Martínez et al., 2025). Lower-level networks move the focus from a broad range of psychological problems to specific processes such as the relations between specific thoughts, motivations, coping mechanisms, and outcomes thereof. For example, two individuals may show the same behavior but the behavior might be differently motivated. Consider going for a run. A person might engage in such behaviour because they are motivated to live a healthy lifestyle. This could lead to positive thoughts, and an improved mood. However, another person might go for a run because they ate one too many cookies, which leads to weight gain related thoughts, which leads to anxiety. The same behaviour is motivated in very different ways, and this difference in motivation could be clinically relevant. These types of networks are valuable because they may be the building blocks of higher-level networks (Wichers et al., 2021). Higher-level networks might be useful to map relevant processes, but not to fully understand them. Zooming in on lower-level networks might be more informative for specific processes and a better guide to the treatment that is needed. Examples of network analysis in practice include feedback (Hall & Luxemburg, 2023), assessment (Scholten & Glombiewski, 2025), case conceptualization (Vogel et al., 2025), and treatment tailoring (Scholten & Glombiewski, 2025; Guðmundsdóttir et al., 2025). The utility of lower-level networks on these settings still needs to be investigated.

Importantly, to ensure the clinical applicability of the present results, future research should be conducted with clinical samples. Although our analyses included individuals reporting some degree of psychopathology (see Jover Martínez et al., 2025), the sample consisted of students rather than patients with clinical-level diagnoses. Replicating these analyses in clinical populations is therefore necessary to establish their relevance in such contexts. In addition, the sample was predominantly female, which may limit the generalizability of the findings. Further research including more male participants is warranted to address this limitation.

## Conclusion

This study investigated the robustness, generalizability, and heterogeneity of psychopathology networks. When using case-dropping bootstrap, temporal and contemporaneous mlVAR networks can be robustly estimated. The generalizability from nomothetic to idiographic networks is fair at a γ = 0.5. This level of generalizability could be due to the heterogeneity in the sample as supported by the presented heterogeneity analyses. However, analyses with a lower γ parameter suggest drastically increased heterogeneity and decreased generalizability. Moving away from clinical diagnoses and finding valid indicators of homogeneous groups is vital to draw conclusions on the generalizability and heterogeneity of network models. To find such groups, a combination of bottom-up clustering approaches, like subgrouped chain graphical VAR (scGVAR) models, and top-down theoretically-driven approaches, like exploring mechanisms underlying psychopathology, is suggested. For this, it is emphasized that single-case research on lower-level networks might be optimal to do such research, and can ultimately contribute to more effective treatments for mental disorders.

## Data Availability

The data that support the findings of this study are available from the authors but restrictions apply to the availability of these data, due to privacy issues, and so are not publicly available. Data are, however, available from the authors upon reasonable request.
